# Molecular Epidemiology of Peste Des Petits Ruminants Virus in West Africa: Is Lineage IV Replacing Lineage II in Burkina Faso?

**DOI:** 10.3390/v16020244

**Published:** 2024-02-03

**Authors:** Abel S. Biguezoton, Guy Sidwatta Ilboudo, Barbara Wieland, Rahinata W-Y. Sawadogo, Fairou F. Dah, Cheick A. K. Sidibe, Adrien Zoungrana, Edward Okoth, Michel Dione

**Affiliations:** 1 Centre International de Recherche-Développement sur l’Elevage en Zone Subhumide (CIRDES), Unité de Recherche Maladies à Vecteurs et Biodiversité (UMaVeB), Bobo-Dioulasso 01 BP 454, Burkina Faso; 2 Animal and Human Health Program, International Livestock Research Institute (ILRI), Ouagadougou 01 BP 1496, Burkina Faso; 3 Institute of Virology and Immunology (IVI), 3147 Mittelhausern, Switzerland; 4 Department of Infectious Diseases and Pathobiology, Vetsuisse Faculty, University of Bern, 3012 Bern, Switzerland; 5 Service Diagnostic et Recherche, Laboratoire Central Vétérinaire (LCV), Bamako BP 2295, Mali; 6 Animal and Human Health Program, International Livestock Research Institute (ILRI), P.O. Box 30709, Nairobi 00100, Kenya; 7 Animal and Human Health Program, International Livestock Research Institute (ILRI), Dakar BP 24265, Senegal

**Keywords:** goat, phylogeny, replacement, morbillivirus, disease, food security

## Abstract

This study aimed at investigating the genetic lineages of peste des petits ruminants virus (PPRV) currently circulating in Burkina Faso. As part of PPR surveillance in 2021 and 2022, suspected outbreaks in different regions were investigated. A risk map was produced to determine high-risk areas for PPR transmission. Based on alerts, samples were obtained from three regions and all sampled localities were confirmed to fall within PPR high risk areas. We collected swab samples from the eyes, mouth, and nose of sick goats. Some tissue samples were also collected from dead animals suspected to be infected by PPRV. In total, samples from 28 goats were analysed. Virus confirmation was performed with RT-PCR amplification targeting the nucleocapsid (N) gene. Partial N gene sequencing (350 bp) was carried out using the RT-PCR products of positives samples to characterise the circulating lineages. Eleven sequences, including ten new sequences, have been obtained. Our study identified the presence of the PPRV lineage IV in the three studied regions in Burkina Faso with a genetic heterogeneity recorded for the sequences analysed. Previously published data and results of this study suggest that PPRV lineage IV seems to be replacing lineage II in Burkina Faso.

## 1. Introduction

Peste des petits ruminants (PPR) are one of the most fatal diseases of small ruminants in Burkina Faso [1]. The disease is caused by a Morbillivirus of the Paramyxoviridae family, related to the viruses responsible for rinderpest, measles, and distemper [2]. The virulence of the virus strain, the species and breed type of the host, the concomitant infection, and the exposure of the animal population prior to infection can influence the severity of clinical signs, morbidity rate, and case fatality rate [2]. When introduced into a naive population, morbidity and mortality can reach almost 100%, causing a major shock to the livelihoods of pastoralists and the small ruminant trade. In Burkina Faso, the economic impact of PPR was estimated between 2014 and 2017 at more than XOF 285 billion (USD 490 million) considering mortalities and morbidities [3]. A modelling study in the country reported that taking into account a 5% shock in the value of small ruminant output to simulate the hypothetical outbreak would reduce the gross domestic product (GDP) at factor cost by 0.62% (i.e., over USD 98 million) considering the GDP in 2019 [4]. Due to its economic and health importance, PPR has been ranked among the priority diseases to be targeted for strict control in Burkina Faso [1,5].

Based on the PPR Monitoring and Assessment Tool (PMAT), Burkina Faso is at the control step of eradication. Its PPR control and eradication plan has been implemented since 2019. PPR surveillance is mainly passive (event-based), based on the suspicion and reporting of cases. Although the disease surveillance is not yet exhaustive, reports suggest that PPR was present in almost all regions of Burkina Faso from 2010 to 2019, with the exception of the Centre and South-West. Furthermore, there were 25 new outbreaks of the disease in 2018 with 52 diagnosed cases and 15 deaths [6]. 

Even if the disease is ranked among the priority diseases in the country, one of the less investigated points on PPR in Burkina Faso is the distribution and circulation of the different viral genetic lineages. Globally, molecular epidemiology of PPR virus (PPRV) led to the identification of four genetically distinct lineages (I, II, III, and IV) and appears to be an effective tool for studying the spread of the virus around the world. Lineage I virus was detected in the 1980s in some West African countries, including Burkina Faso [7]. Recent studies have confirmed the presence of lineage II in West and Central Africa, while lineage III is common to East Africa and the southern part of the Middle East. Lineage II has also been reported in Burkina Faso based on samples collected in 2008 [8]. In Asia, only lineage IV viruses have been detected, and this lineage has spread rapidly to the Middle East. Recently, lineage IV viruses have been regularly reported in various African countries, and this lineage is becoming also the predominant lineage in West Africa [9]. The first report of lineage IV of PPRV in Burkina Faso is related to a sampling carried in December 2021 by Couacy-Hymann et al. [10]. These authors have also published sequences of lineage II coming from samples collected in February 2019 [10]. Such results suggest a potential co-circulation of both lineages in Burkina Faso. This study aimed to strengthen and adding value to ongoing surveillance by investigating currently circulating lineages of PPRV in Burkina Faso.

## 2. Materials and Methods

This study was linked to ongoing surveillance of PPR in Burkina Faso from March 2021 to October 2022 based on field veterinary service alerts. While efforts have been made to cover all the 13 regions of the country for sampling, alerts (leading to sampling) came only from three regions. Sampling was thus carried out in Plateau-Central, Centre-Ouest, and Cascades. A risk map based on key epidemiological factors for PPR was produced to determine if the locality where samples came from corresponded (or not) to high-risk areas for PPR transmission.

### 2.1. PPR Transmission Risk Map 

To determine whether the sampling municipalities correspond to high-risk areas for PPR transmission, a risk map was produced using the qualitative and mapped risk analysis method in QGIS version 3.22. For this purpose, parameters such as accessibility; small ruminant density; animal mobility; markets (related to animals); watering points; and livestock tracks have been considered. The ranking of variables of livestock markets and those of watering points has been done by three experts including one from national veterinary service. Regarding accessibility, values have been dividing in four classes of quantile in QGIS, considering the fact that lower is the accessibility value higher is the risk. While for the small ruminant density the same approach of quantile division was applied the sense of the risk was different. In other words, the lower the density, the lower the risk. Animal mobility data were used to perform social network analysis (SNA). The resulting “outdegree” and “betweenness” values were then dividing in four classes of quantile in QGIS too. The presence of livestock tracks, and international markets received the very high-risk score. For the other livestock markets, very high and high-risk scores were applied according to their size or role in the animal distribution (grouping and exports vs. production and consumption). For watering points, according to the type of watering points three categories have been set: very high, high, and low-risk points. The risk map was then produced in QGIS with the four risk levels of PPR distribution: i.e., very-high, high, low, and negligible.

### 2.2. Sampling 

Samples were collected during PPR disease surveillance from March 2021 to October 2022. During that time, alerts from field veterinary service of five outbreaks from October 2021 to May 2022 in four municipalities (i.e., Banfora (#4); Koudougou (#3); Niangoloko (#2); Niou (#1)) belonging to three provinces in three regions (Table 1) were received.

The clinical signs based on which the sampling has been carried out were bilateral nasal discharge, cough and/or diarrhoea with or without bilateral lacrimation, sneezing, dyspnoea, or erosions of the mouth. In each of the five outbreaks, samples were collected from six animals including three swab samples (eye, nasal, mouth). Six organs (liver, spleen, lung, pharyngeal ganglion, bronchial ganglion, mesenteric ganglion) were also collected from dead animals suspected to be infected by PPRV. The swabs from each animal were first used in the field to confirm presence of PPRV using the RAPID test PPR (IDVET) according to manufacturer recommendations (Table 1). In total, 96 samples were collected from 28 live and two dead goats suspected of being infected with PPRV. However, due to poor field transport logistics including bad roads and storage problems only 74 samples, 27 from live samples and one from a dead goat were suitable for PPRV diagnostic (Table 1). 

### 2.3. RT-PCR and Sequencing 

Prior to PCR-screening, RNA extraction was carried out using the QIAGEN RNeasy Mini kit, according to manufacturer (QIAGEN, Hilden, Germany). Virus confirmation was then performed with RT-PCR amplification targeting the nucleocapsid (N) gene fragment of 350 bp with primers NP3 and NP4 [11] using the Qiagen one-step RT-PCR kit (QIAGEN, Hilden, Germany) according to manufacturers instructions. 

Seven microliters of each PCR product were then subjected to electrophoresis on a gel concentrated at 1.5%. The products of positives samples were sequenced in both forward and reverse sense by AZENTA (Griesheim, Germany) based on Sanger’s method. 

### 2.4. Sequences and Phylogenetic Analyses

Sequence analyses included consensus sequence building with BioEdit version 7 [12]. Using BLASTn, pairwise alignment allowed comparison of current study sequences with those that have been published on GenBank. 

Phylogenetic analyses were performed using sequences of 231 bp of samples of this study and reference sequences of the four lineages (I, II, III, and IV) previously published. The reduction of the size of the sequence amplified by RT-PCR (i.e., 350 bp) was necessary to include the most representative reference sequences previously published. A phylogenetic tree was then constructed using the maximum likelihood method [13] with MEGA version 6 [14]. The model Kimura 2-parameter with a discrete Gamma distribution (K2+G) with five rate categories were applied as nucleotide substitution model. For the robustness of the analyses, 10,000 bootstrap replications were applied. Selection of reference sequences from other countries was based on previous published articles in Africa (focusing on West Africa) but also on the sequences matching found on GenBank. Efforts have been made to limit sequences with 100% matching in the phylogenetic tree.

## 3. Results

**Figure 1:**
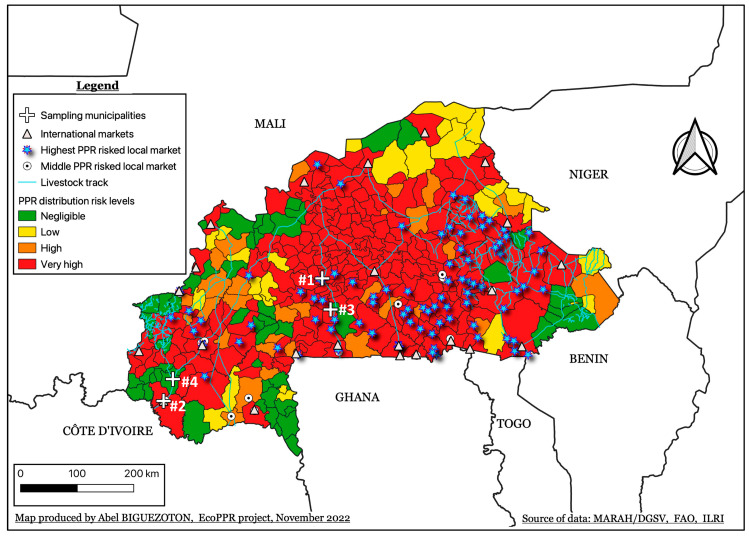
PPR risk distribution map and sampling sites in Burkina Faso. Site #1 refers to Niou, while #2 corresponds to Niangoloko. #3 is Koudougou while #4 is Banfora.

The risk map results revealed that central Burkina Faso has the largest area with a very-high risk of PPR transmission (Figure 1). However, northern and southern parts of the country also contain with very high-risk of PPR distribution, albeit to a lesser extent. Projecting the sampling municipalities on the risk map, pointed out that all of them, except site #4, are located in areas of very high-risk transmission of PPR (Figure 1). 

Forty-two (42) of the 74 examined samples tested positive by RT-PCR for PPRV (Table 1). These samples were from 22 goats. Among these positive samples, 20 RT-PCR products of 20 goats (one sample per goat) were selected based on electrophoresis analysis for sequencing. Only sequences of 13 samples were clean enough and thus suitable for analyses. However, due two 100% matching between some of them only 11 sequences were used for further comparative analyses. Among them, 10 sequences were not 100% identical with existing sequences in GenBank and thus they have been published in GenBank with accession numbers from OR634800 to OR634809 (Table 1).

Based on the 231 bp sequences used for phylogenetics analyses, the 11 studied sequences showed 98–100% nucleotide identity with other lineage IV sequences published in GenBank. All of these sequences are from West Africa (i.e., MW281026, MW600928, etc.). 

**Figure 2:**
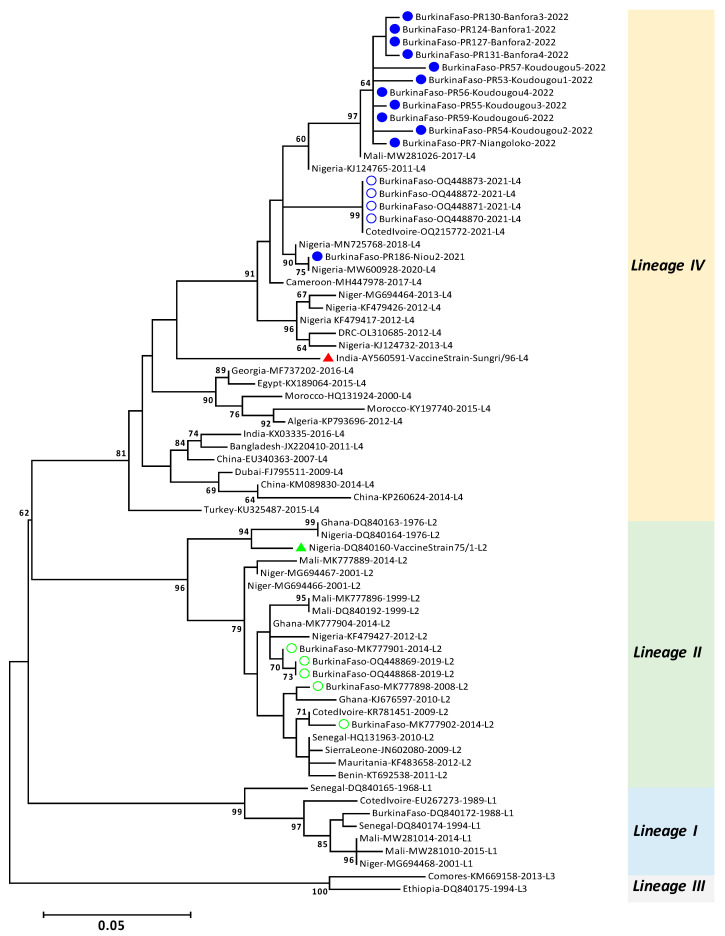
Maximum likelihood phylogenetic relationships based on N gene sequences (231 bp). Bootstrap value < 60% have been removed. Blue filled circles refer to samples of this study. Blue empty circles correspond to lineage IV published samples from Burkina Faso whereas green empty circles refer to lineage II published samples for the same country. Red triangle corresponds to vaccine strain from lineage IV while the green one refers to vaccine strain from lineage II. L1, L2, L3, and L4 refer to lineages I, II, III, and IV, respectively.

Moreover, phylogenetic analyses revealed that all sequences obtained in this study belonged to lineage IV (Figure 2). Comparing all lineage IV sequences, the phylogenetic tree highlighted two groups supported by a bootstrap value of 81%. One group contained sequences from West and Central Africa and the second group gathered sequences from North Africa, Middle East, and Asia. Our sequences belonged to the group of West and Central Africa and were separated into two subgroups, one gathering sequences of site #1 with a sample from Nigeria and the other one regrouped sequences of sites #2, #3, and #4 with a sample from Mali (Figure 2). None of the lineage IV sequences in this study grouped with previous published lineage IV sequences from Burkina Faso.

## 4. Discussion

Outbreak investigation through a well-established disease surveillance system enabled us to investigate lineages of PPRV circulating in different regions of Burkina Faso. The study covered three of the thirteen regions where livestock, especially small ruminants, is important to the communities. The study did not exclusively focus on the surveillance of these regions, but no alerts from the other regions were received during the period of the investigation. However, the investigated regions (mainly the regions corresponding to sites Koudougou (#3), Niangoloko (#2), and Niou (#1)) are PPR high risk areas as shown by the risk map. Considering high animal mobility in the country [15,16], there is high potential of PPRV spread not only within these regions but also between them and even with bordering countries including Côte d’Ivoire, Ghana, and Mali. It is important to note that PPRV positive samples from animals within the same mobility areas or communities are significantly more likely to belong to the same genetic clade [17]. In other words, they are more likely to be genetically similar or to belong to the same lineage. The fact that all samples in this study belonged to lineage IV and that the origin of the samples suggests wide circulation of lineage IV in Burkina Faso. A recent publication has also evidenced the presence of this lineage IV in Burkina Faso [10]. Samples analysed by the authors have been collected in December 2021 at Loumbila in Plateau Central region while we carried out sampling for the first outbreak event of our study in October 2021 at Niou in the same region (Table 1). Thus, non-published data related to the presence of lineage IV of PPRV in Burkina Faso existed before the data published by Couacy-Hymann et al. [10]. Despite this, the exact date of the first introduction of lineage IV of PPRV in Burkina Faso is not known. However, the similarity of most of our sequences with the sequence MW281026, evidenced in Mali in 2017 in addition with the genetic diversity within our sequences suggest that lineage IV likely has been present in Burkina Faso for several years. 

The presence of lineage IV of PPRV in Burkina Faso confirmed the continuous spread of this lineage in West Africa [8]. However, the 11 sequences resulting from the current study did not group with previously identified PPRV from Burkina Faso in the phylogenetic tree (Figure 2). They belonged to two subclades involving other samples from West Africa. The clade containing the sequences belonging to this study is supported by a bootstrap value of 97 and that containing previously published sequences is supported by bootstrap value of 99. However, the large clade containing all the lineage IV viruses from Burkina Faso is supported by a bootstrap value of <60 (Figure 2). This heterogeneity of PPRV circulating within a country has been reported in the past [18,19,20]. Considering the subclades of the phylogenetic tree including sequences of our study and those of Couacy-Hymann et al. [10], the presence of the lineage IV in Burkina Faso likely came from at least three independent introductions from three different countries: i.e., Côte d’Ivoire, Mali, and Nigeria. However, such conclusions require further analyses with more robust approaches like the full genome sequencing combined with qualitative analysis and mapping of the risks of introduction of PPR and more sampling locations covering all the country with more attention to the international markets and between country borders. 

Interestingly, site #1 (Niou) in central Burkina Faso represents is also a hot spot of small ruminant mobility in the country, connecting the North to the South. After the first outbreak event recorded in October 2021, we also found lineage IV positive samples in sites #2 to #4 (from March to May 2022) confirming the statement above related to the link between animal mobility and genetic data of virus samples [17]. However, given the results of the phylogenetic analyses and data we had, we could not conclude that sequences from the three other sites come from the evolution of the sequences from site #1 (Niou). 

The genetic lineage II was detected until 2019 in the region of Centre Ouest of Burkina Faso [10], which also includes site #3 (Koudougou) of this study. However, all six samples collected there in this study in March 2022 belong to lineage IV. The small size of the sampling can be considered as a limiting factor to study the diversity of lineages circulating in the region. Nevertheless, given the movement of the small ruminants, lineage II likely would have been detected were it still circulating. Indeed, the results of this study suggest that the lineage II of PPRV is being replaced by lineage IV in Burkina Faso, although further investigations are needed to confirm it. The mechanism supporting such replacement is not yet clearly described. Nevertheless, the fact that among the four circulating lineages in the world, lineages II and IV are the most similar [21] can be one of the parameters allowing this replacement. Added to this, some specific characteristics of lineage IV (e.g., better capacity for replication, transmission, etc.) would favour this replacement. Surprisingly, it is not the first time such replacement would be observed. For instance, in Ethiopia the replacement of lineage III by lineage IV has been reported [22,23]. As well in West Africa, lineage IV is becoming the predominant viral lineage of PPRV, replacing previously circulating lineages I and II [10]. How and why lineage IV is becoming the predominant lineage in West Africa is unclear [9]. Investigations are also needed to determine whether the virus is more or less pathogenic than viruses of other lineages circulating previously in a considered country or transboundary countries. Fortunately, the vaccine based on PPRV/Nigeria/75/1 (lineage II) which is the most used in West African countries, has a complete cross-protective ability against virulent PPRV strains from the four known lineages of the virus [21]. Nonetheless, there is a need for a strong surveillance system in place to monitor the spread of the PPRV for better control of PPR in West Africa. 

## 5. Conclusions

This study confirms the presence of the PPRV lineage IV in Burkina Faso with a genetic heterogeneity of the sequences analysed indicating repeated introductions and likely circulation for several years. Based on our analysis, lineage IV seems to be increasingly replacing lineage II. These findings require further investigations to cover the 13 regions of the country to better understand how PPRV lineages have spread in the country and if lineage II is still present and what the implications for PPR control are. 

The results of this study demonstrate that genotyping is a useful addition to conventional surveillance activities and provide additional important insights on how disease move within a country and between countries. Better understanding of genotypes distribution overtime helps to better target control of PPR in Burkina Faso and in the Sahel. 

## Figures and Tables

**Table 1:** Sample description. Sample result from five outbreak events from October 2021 to May 2022.

Region	Province	Location	Outbreak Events	Sample ID	Animal	Animal Type	Sample Type	Collection Date	RT-PCR_PPRV Results	Successful Sequencing	Accession Numbers
Plateau-Central	Kourweogo	Niou	1	PR20	1	Goat	Eye swab	26 October 2021	+	No	
				PR22			Nasal swab		+	No	
				PR24			Mouth swab		+	No	
				PR25	2		Eye swab		+	No	
				PR27			Nasal swab		-		
				PR29			Mouth swab		+	No	
				PR31	3		Eye swab		-		
				PR33			Nasal swab		-		
				PR35			Mouth swab		-		
				PR38	4		Eye swab		-		
				PR40			Nasal swab		-		
				PR42			Mouth swab		-		
				PR44	5		Eye swab		+	No	
				PR46			Nasal swab		-		
				PR48			Mouth swab		-		
				PR185	6		Lung		+	Yes	100% matching with MW600928
				PR186			Spleen		+	Yes	
				PR187			Pharyngeal ganglion		+	No	
				PR188			Bronchial ganglion		+	No	
				PR189			Mesenteric ganglion		+	No	
Cascades	Comoe	Banfora	2	PR106	1	Goat	Eye swab	23 December 2021	-		
				PR107			Nasal swab		+	No	
				PR108			Mouth swab		-		
				PR109	2		Eye swab		-		
				PR110			Nasal swab		+	No	
				PR111			Mouth swab		+	No	
				PR112	3		Eye swab		+	No	
				PR113			Nasal swab		-		
				PR114			Mouth swab		+	No	
				PR115	4		Eye swab		+	No	
				PR116			Nasal swab		-		
				PR117			Mouth swab		-		
Cascades	Comoe	Niangoloko	3	PR1	1	Goat	Eye swab	8 March 2022	-		
				PR2			Nasal swab		+	No	
				PR3			Mouth swab		-		
				PR4	2		Eye swab		-		
				PR5			Nasal swab		-		
				PR6			Mouth swab		-		
				PR7	3		Eye swab		+	Yes	OR634808
				PR8			Nasal swab		+	No	
				PR9			Mouth swab		+	No	
				PR10	4		Eye swab		-		
				PR11			Nasal swab		-		
				PR12			Mouth swab		-		
				PR13	5		Eye swab		-		
				PR14			Nasal swab		-		
				PR15			Mouth swab		-		
				PR16	6		Eye swab		-		
				PR17			Nasal swab		+	No	
				PR18			Mouth swab		-		
Centre Ouest	Boulkiemde	Koudougou	4	PR49	1	Goat	Eye swab	15 March 2022	+	No	
				PR51			Nasal swab		-		
				PR53			Mouth swab		+	Yes	OR634804
				PR54	2		Eye swab		+	Yes	OR634806
				PR55	3		Nasal swab		+	Yes	OR634800
				PR56	4		Nasal swab		+	Yes	OR634805
				PR57	5		Nasal swab		+	Yes	OR634801
				PR58	6		Mouth swab		-		
				PR59			Mouth swab		+	Yes	OR634803
Cascades	Comoe	Banfora	5	PR118	1	Goat	Eye swab	7 May 2022	-		
				PR119			Nasal swab		-		
				PR120			Mouth swab		-		
				PR121	2		Eye swab		+	No	
				PR122			Nasal swab		+	No	
				PR123			Mouth swab		+	No	
				PR124	3		Eye swab		+	Yes	OR634809
				PR125			Nasal swab		+	No	
				PR126			Mouth swab		+	No	
				PR127	4		Eye swab		+	Yes	OR634809
				PR128			Nasal swab		+	No	
				PR129	5		Mouth swab		+	No	
				PR130			Eye swab		+	Yes	OR634802
				PR131	6		Nasal swab		+	Yes	OR634807
				PR132			Mouth swab		+	No	

“+” refers to positive results of RT-PCR whereas “-“ corresponds to negative results.

## Data Availability

The PPRV N gene partial sequences generated by this study are available online in Genbank through the accession numbers: OR634800 to OR634809.
